# Tissue expression of retinoic acid receptor alpha and CRABP2 in metastatic nephroblastomas

**DOI:** 10.1186/s13000-018-0686-z

**Published:** 2018-01-22

**Authors:** Ana Paula Percicote, Gabriel Lazaretti Mardegan, Elizabeth Schneider Gugelmim, Sergio Ossamu Ioshii, Ana Paula Kuczynski, Seigo Nagashima, Lúcia de Noronha

**Affiliations:** 1 Federal University of Paraná, Curitiba, Brazil; 2 Anatomic Pathology Service at the Pequeno Príncipe Hospital, Curitiba, Brazil; 3 Department of Medical Pathology, Federal University of Paraná and School of Health of the Pontifical Catholic University of Paraná, Curitiba, Brazil; 4 Oncology Service at the Pequeno Príncipe Hospital, Curitiba, Brazil; 5 School of Health of the Pontifical Catholic University of Paraná, Curitiba, Brazil

**Keywords:** Nephroblastoma, Retinoic acid, CRABP2

## Abstract

**Background:**

Nephroblastoma or Wilms tumor is the most frequent kidney cancer in children and accounts for 98% of kidney tumors in this age group. Despite favorable prognosis, a subgroup of these patients progresses to recurrence and death. The retinoic acid (RA) pathway plays a role in the chemoprevention and treatment of tumors due to its effects on cell differentiation and its antiproliferative, anti-oxidant, and pro-apoptotic activities. Reports describe abnormal cellular retinoic acid-binding protein 2 (CRABP2) expression in neoplasms and its correlation with prognostic factors and clinical and pathological characteristics. The aim of this study was to evaluate the immunohistochemical expression of retinoic acid receptor alpha (RARA) and CRABP2 in paraffin-embedded samples of nephroblastomas via semiquantitative and quantitative analyses and to correlate this expression with prognostic factors.

**Methods:**

Seventy-seven cases of nephroblastomas were selected from pediatric oncology services. The respective medical records and surgical specimens were reviewed. Three representative tumor samples and one non-tumor renal tissue sample were selected for the preparation of tissue microarrays (TMA). The Allred scoring system was used for semiquantitative immunohistochemical analyses, whereas a morphometric analysis of the stained area was employed for quantitative evaluation. The nonparametric Mann-Whitney test was used for comparisons between two groups, while the nonparametric Kruskal-Wallis test was used to compare three or more groups.

**Results:**

Immunopositivity for RARA and CRABP2 was observed in both the nucleus and cytoplasm. All histological components of the nephroblastoma (blastema, epithelium, and stroma) were positive for both markers. RARA, based on semiquantitative analyses, and CRABP2, bases on quantitative analyses, exhibited increased immunohistochemical expression in patients with metastasis, with *p* values of 0.0247 and 0.0128, respectively. These findings were similar to the results of the quantitative analysis of RARA expression, showing greater immunopositivity in tumor samples of patients subjected to pre-surgical chemotherapy. No significant correlation was found with the other variables studied, such as disease stage, anaplasia, risk group, histological type, nodal involvement, and clinical evolution.

**Conclusions:**

Semiquantitative and quantitative analyses of the markers RARA and CRABP2 indicate their potential as biomarkers for tumor progression and their participation in nephroblastoma tumorigenesis.

**Electronic supplementary material:**

The online version of this article (10.1186/s13000-018-0686-z) contains supplementary material, which is available to authorized users.

## Background

Nephroblastoma or Wilms tumor is the most frequent kidney cancer in children and accounts for 98% of kidney tumors in this age group [1]. Nephroblastoma originates in metanephric blastema cells that histologically resemble the undifferentiated blastema, stroma, and primitive renal tubular structures of the fetal kidney [2]. The prognosis of patients with this cancer improved dramatically after the formation of cooperative groups. The development of treatment protocols resulted in global survival rates above 90% [3].

Despite this therapeutic success, prognostic factors such as lymph node metastases, anaplastic histology, bilateral disease, molecular characteristics, stage III/IV disease, tumor rupture, and renal vein and inferior vena cava thrombi are associated with relapse and reduced survival [3–5].

In a frantic search for new therapeutic targets and for a better understanding of the molecular pathways responsible for tumorigenesis and tumor progression in nephroblastoma, retinoic acid (RA) emerged as a therapeutic alternative for the treatment of this cancer. Numerous genes involved in the RA signaling pathway seem to participate in the progression and cellular differentiation of nephroblastoma [6, 7].

RA is the most active metabolite of vitamin A, and its function is essential for many biological processes, such as fetal development and the proliferation, differentiation and apoptosis of normal and tumor cells [8]. The effects of RA on the regulation of gene expression depend on intracellular RA distribution secondary to the concentration of intracellular proteins such as cellular retinoic acid-binding protein 2 (CRABP2) and to the activity on RA receptors, designated retinoic acid receptors (RARs) and retinoid X receptors (RXRs) [8–10]. Retinoic acid receptor alpha (RARA) is a ligand-dependent transcription factor that regulates target gene expression after RA binding [8]. RA can be used for the chemoprevention and treatment of tumors due to its effects on cell differentiation and its antiproliferative, anti-oxidant and pro-apoptotic activities [11, 12]. CRABPs are low-molecular-weight, intracellular proteins that act on RA-induced transcriptional activity, maintaining an adequate RA metabolism [13].

Previous studies have described changes in the RA pathway in nephroblastomas [6, 7, 14]. The overexpression of CRABP2 is related to advanced stages of this cancer, which indicates the involvement of the RA pathway in tumor progression [7, 15, 16]. CRABP2 protein expression has been observed in human fetal kidney samples during mesenchymal-epithelial transition and in the blastemal component of nephroblastoma; in the latter, the RA pathway was found to be associated with CRABP2 upregulation [2].

This study aims to evaluate the distribution of RARA and CRABP2 protein expression and positivity in nephroblastoma tissue specimens and to correlate these results with clinical and pathological prognostic factors.

## Methods

### Patients

We selected 77 patients diagnosed with nephroblastoma between 1994 and 2012 who were treated at pediatric oncology services of the Clinics Hospital Complex/Federal University of Paraná, Pequeno Príncipe Children’s Hospital and Erasto Gaertner Hospital. This study was approved by the ethics committees of the three institutions.

Hematoxylin-eosin-stained histological slides were reviewed and classified according to the histological type and presence of anaplasia. Relevant clinical data, such as the gender of patients, age at diagnosis, initial clinical presentation, disease stage, risk group, presence of metastasis, nodal involvement, treatment received, and clinical evolution, were obtained.

Twelve tissue microarrays (TMA) containing samples representative of the tumors were constructed. For each case, three distinct tumor areas and one sample of non-tumor renal tissue were selected to construct the TMA.

Information regarding the clinical evolution, initial treatment (chemotherapy or surgery), and prognostic factors, such as disease stage, presence of metastasis, histological type, presence of anaplasia, risk group, and nodal involvement, was compiled for statistical analysis and comparison with the immunoexpression of markers in tumor samples.

### Immunohistochemistry

Histological sections of 4 μm thickness were obtained for immunohistochemistry. The slides were then subjected to deparaffinization, dehydration, and rehydration. Endogenous peroxidase was blocked using methyl alcohol and hydrogen peroxide for the first blocking step and distilled water and hydrogen peroxide for the second blocking step. The next step was incubation with primary antibodies chosen for the study, including rat anti-RARA monoclonal antibody (clone 2C9-1F8; 1:800 dilution, Abcam, Cambridge, MA, USA) and rabbit anti-CRABP2 polyclonal antibody (1:50 dilution, Bioss, Woburn, MA, USA), for one hour in a humid chamber at room temperature. Secondary antibody (Advance™ HRP Dako®, Dako Corporation, Carpinteria, CA, USA) conjugated with the polymer dextran was incubated with the slides for 30 min at room temperature. For staining, the diaminobenzidine (DAB) + substrate complex (buffer for DAB dilution) was added to the slides, which were counterstained with Harris hematoxylin and subsequently dehydrated with 100% ethanol and clearing with analytical grade xylol. The stained slides were mounted in histological resin for microscopy (Entellan, Merck®). Positive and negative controls were included in all reactions [17].

### Evaluation of immunoexpression

Immunohistochemical expression was evaluated through semiquantitative and quantitative analyses of both nuclear and cytoplasmic staining. For the semiquantitative analysis, the Allred scoring system was used, which consists of scores of the proportion and intensity of both nuclear and cytoplasmic staining that are summed to give the total Allred score. The proportion score was calculated as the ratio between positive cells and the total number of cells and was classified as 0 (0%), 1 (> 0–1%), 2 (≥1%–10%), 3 (> 10%–33%), 4 (> 33%–66%), and 5 (> 66%–100%). The intensity score was classified as 0 (negative), 1+ (weak), 2+ (moderate), and 3+ (strong). The total score was calculated as the sum of the proportion and intensity scores and ranged from 0 to 8 [18].

Quantitative morphometric analysis was performed using images obtained by a Zeiss Axioscan Slide Scanner (Germany) at 20× magnification, which generates digital tagged image file format (TIFF) files. After scanning, the software generated images in the photomicrograph format, which were analyzed using the image analysis software Image-Pro Plus® (Rockville, MD, USA). The immunostained area of each photomicrograph was measured, including both nuclear and cytoplasmic areas, and was expressed in square micrometers, and the mean was calculated for each case and transformed into percentage by half-magnification field (HMF) by dividing by the constant 115.226,1 μm^2^ and multiplying by 100 for subsequent statistical analysis [19].

### Statistical analysis

For the evaluation of the data obtained, quantitative variables were expressed as the means, medians, minimum values, maximum values, and standard deviations. Qualitative variables were expressed as frequencies and percentages. For comparisons between two groups, the non-parametric Mann-Whitney test was used for quantitative variables. Three or more groups were compared using the non-parametric Kruskal-Wallis test. The normality of the variables was evaluated by the Shapiro-Wilk test. Pearson correlation test was applied to measure the dependence of two variables. Values ​​of *p* < 0.05 indicated statistical significance. The data were analyzed using IBM SPSS Statistics v. 20 software.

## Results

### Population characteristics

Of the total 77 selected patients with nephroblastoma, the mean age at diagnosis was 33.6 months (0–108 months). The main clinical findings related to diagnosis were increased abdominal girth in 40.2% (*n* = 31), abdominal mass in 37.7% (*n* = 29), abdominal pain in 11.7% (*n* = 9), fever in 10.4% (*n* = 8), vomiting in 3.9% (*n* = 3), and weight loss in 3.9% (*n* = 3) of the patients. As an initial treatment, 67.1% of the patients underwent pre-surgical chemotherapy, while 25% underwent surgery. Data regarding the initial treatment of one patient could not be retrieved. The clinical and demographic data are summarized in Table 1.

**Table 1:** Patients’ baseline characteristics

Variable	Value
Gender (female:male)	31:46
Age (months), median	26
Metastases, n (%)	
Yes	17 (22.4)
No	59 (77.6)^a^
Histological risk group, n (%)	
High risk	9 (11.7)
Intermediate risk	68 (88.3)
Lymph nodes, n (%)	
Negative	36 (46.8)
Positive	4 (5.2)^b^
Local stage, n (%)	
I	47 (64.4)
II	13 (17.8)
III	13 (17.8)^c^
Histological classification, n (%)	
Nephroblastoma - epithelial type	10 (13.0)
Nephroblastoma - stromal type	9 (11.7)
Nephroblastoma - mixed type	37 (48.1)
Nephroblastoma - regressive type	4 (5.2)
Nephroblastoma - blastemal type	12 (15.6)
Nephroblastoma - diffuse anaplasia type	5 (6.5)
Clinical evolution, n (%)	
Disease-free	57 (74.0)
Dead	11 (14.3)^d^

^a^data could not be retrieved; ^b^remaining patients did not undergo lymph node resection; ^c^specimens with impaired staging; ^d^patients lost to follow-up or transferred to another center during the study

### Immunohistochemical analysis

**Fig. 1:**
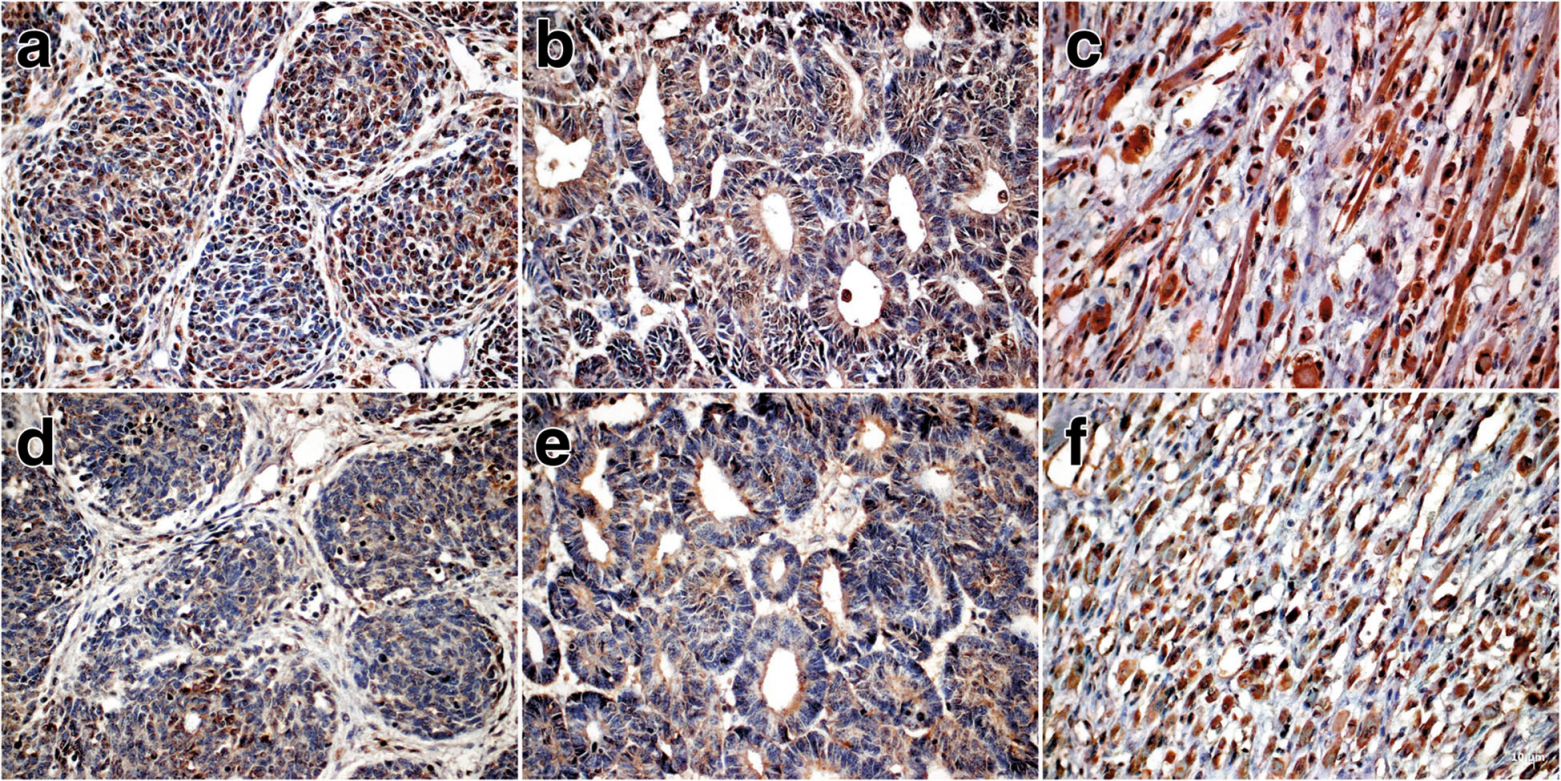
Immunohistochemical expression of RARA and CRABP2 in nephroblastoma samples. RA immunoexpression: **a**, blastema; **b**, epithelium; **c**, stroma; CRABP2 immunoexpression: **d**, blastema; **e**, epithelium; **f**, stroma (40×)

Immunopositivity for RARA and CRABP2 was observed in all three histological components of nephroblastoma (blastema, stroma, and epithelium). Cytoplasmic and nuclear positivity for CRABP2 was present in 100% and 56% of the samples, respectively. A total of 94.7% of the cases were positive for RARA in both the nucleus and cytoplasm. The total Allred score ranged from 3 to 7 for both markers, with a median of 6 for RARA and 5 for CRABP2 (Fig. 1).

The immunoexpression of RA and CRABP2 was not restricted to a specific histological type or histological component. Areas with rhabdomyoblastic differentiation and anaplastic nephroblastomas expressed both markers.

The results obtained by quantitative analysis of RARA and CRABP2 expression in the nephroblastoma samples showed median values of 24.2% per HMF (6.3%–40.3%) and 26.3% per HMF (4.2%–40.4%), respectively (Table 2).

**Table 2:** Median immunoexpression of RARA and CRABP2 in nephroblastomas based on histological subtype

Variable	Semiquantitative, median (minimum:maximum)				Quantitative analysis (%), median (minimum:maximum)			
	RARA	*p* value	CRABP2	*p* value	RARA	*p* value	CRABP2	*p* value
Metastases								
Yes	6 (4–7)	0.9428	5 (3–7)	0.0128	27.1 (17.3–33.8)	0.0247	28.7 (13.6–36.5)	0.0844
No	6 (3–7)		4 (0–7)				25.4 (4.2–40.4)	
Histological risk group								
High risk	5.5 (4–7)	0.7591	4 (3–7)	0.5615	20.0 (12.4–31.7)	0.6014	24.6 (18.9–32.3)	0.8628
Intermediate risk	6 (3–7)		5 (0–7)		24.6 (6.3–40.3)		26.2 (4.2–40.4)	
Lymph nodes								
Negative	6 (3–7)	0.3291	4.5 (0–6)	0.7087	24.6 (6.3–35.7)	0.8715	26.8	0.9197
Positive	6 (5–7)		5 (3–6)		23.2 (19.4–29.1)		25.7	
Local stage								
I	6 (3–7)	0.3425	5 (3–7)	0.8520	25.3 (6.3–40.3)	0.0525	26.2	0.4731
II	6 (5–7)		5 (0–7		20.7 (7.3–33.6)		23.4	
III	5 (4–7)		4 (3–6)		20.2 (12.4–29.1)		26.8	
Histological classification								
Nephroblastoma - epithelial type	6 (5–6)	0.8286	5 (3–7)	0.5188	27.0 (19.9–34.4)4.6	0.2300	25.9 (15.2–39.5)27.6	0.6782
Nephroblastoma - stromal type	5 (5–7)	5 (3–6)			24.3 (14.7–29.0)1		21.4 (6.2–40.4)28.4	
Nephroblastoma - mixed type	6 (3–7)	5 (3–6)			24.4 (6.3–35.6)23.6		26.9 (4.2–37.4)-7.3	
Nephroblastoma - regressive type	6 (5–7)	5 (4–6			26.4 (20.0–31.8)5		27.2(15.2–38.5)6	
Nephroblastoma - blastemal type	6 (4–7)	5 (0–5)			24.920.9 (17.3–40.3)		28.2 (15.1–37.0)27.5	
Nephroblastoma - diffuse anaplasia type	6 (4–7)	4.5 (3–7)			14.821.3 (12.4–31.8)		2.24 (20.0–28.3)4.7	
Overall nephroblastoma	6 (3–7)	5 (0–7)			24.2 (6.3–40.3)		26.3 (4.2–40.4)	
Overall non-tumor renal parenchyma	6 (5–8)	6 (4–7)			30.4 (1.4–49.7)		33.8 (0.6–52.0)	

The immunoexpression of RARA and CRABP2 was also observed in samples of renal parenchyma. Glomeruli and renal tubules expressed RARA and CRABP2 both in the nucleus and cytoplasm (Additional file 1: Figure S1). The median values of the total Allred score obtained for renal parenchyma samples were 6 for RARA and 5 for CRABP2 (Fig. 1).

### Prognostic importance

The results obtained through quantitative and semiquantitative analyses were compared to clinical and pathological prognostic factors such as histological type, disease stage, risk group, presence of metastasis, anaplasia, clinical evolution, nodal involvement, and pre-surgical chemotherapy.

**Fig. 2:**
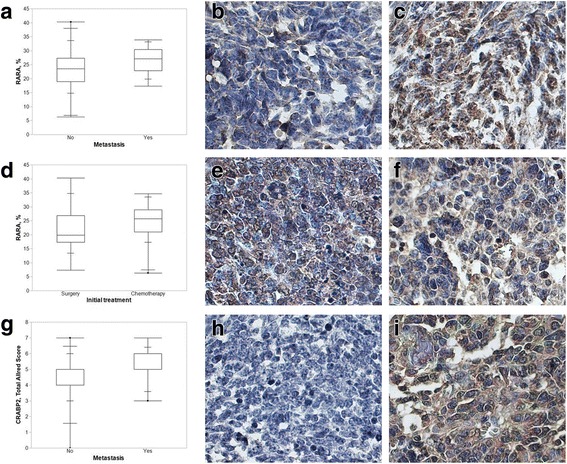
**a** Quantitative analysis of RARA immunoexpression as a function of the presence of metastasis. The box-plot represents the results of the quantitative analysis of RARA immunoexpression according to the presence of metastasis, showing RARA immunoexpression in nephroblastomas with and without metastasis. Increased immunopositivity was observed in patients with metastasis (*p* = 0.0247). **b** and **c** Immunohistochemical expression of RARA in nephroblastomas without (**b**) and with metastasis (**c**) (63×). **d** Quantitative analysis of RARA immunoexpression as a function of the initial treatment. The box-plot represents the results of the quantitative analysis of RARA immunoexpression according to the type of initial treatment, showing RARA immunoexpression in nephroblastoma samples from patients subjected to pre-surgical chemotherapy and patients subjected to surgery as initial treatment (*p* = 0.0330). **e** and **f** Immunohistochemical expression of RARA in nephroblastoma samples from patients subjected to surgery (**e**) and pre-surgical chemotherapy (**f**) (63×). **g** Semiquantitative analysis of CRABP2 immunoexpression as a function of the presence of metastasis. The box-plot represents the results of the quantitative analysis of CRABP2 immunoexpression according to the presence of metastasis, showing CRABP2 immunoexpression in nephroblastomas with and without metastasis. Increased immunopositivity was observed in patients with metastasis (*p* = 0.0128). **h** and **i** Immunohistochemical expression of CRABP2 in nephroblastoma without (**h**) and with metastasis (**i**) (63×)

According quantitative analyses, the protein expression of RARA was increased in patients with metastasis (*p* = 0.0247) and in patients who underwent pre-surgical chemotherapy (*p* = 0.0330) (Fig. 2).

The semiquantitative analysis for CRABP2 indicated higher expression in patients with metastasis (*p* = 0.0128) (Fig. 2). This group of patients, with scores 6 and 7 in semiquantitative analysis, were mostly of intermediate risk (77.8%), without lymph node metastasis (75.0%), with histologically mixed type tumors (53.3%), with local stage I disease (66.7%) and free of disease (86.7%).

Additional evaluation did not demonstrate association between initial distant metastasis and treatment (*p* = 0.1288), local stage (*p* = 0.8700), risk group (*p* = 0.9911) or nodal involvement (*p* = 0.3628). The results were similar for the analysis between local stage and treatment (*p* = 0.9034).

No significant correlation was found between RARA and CRABP2 immunopositivity and clinical evolution, disease stage, anaplasia, nodal involvement, risk group, or histological type (*p* > 0.05).

## Discussion

The present work is the first report of the evaluation of RARA and CRABP2 immunoexpression as potential biomarkers and therapeutic targets in nephroblastomas.

The essential functions of RA in biological processes, such as differentiation, proliferation, and apoptosis, have prompted the evaluation of this protein that had not been previously studied in nephroblastomas [8]. The effects of RA depend on intracellular proteins, such as CRABP2, that maintain an adequate RA metabolism by increasing its availability and transporting it to the nucleus [10, 13, 20]. The effects of RA on gene expression are mediated by nuclear receptors, such as RARA [8]. RARA can be translocated from the cytoplasm to the nucleus after being synthesized, modified and stimulated by RA. Under physiological conditions, RARA is located in the cytoplasm and nucleus [21]. RARA gene mutations observed in promyelocytic leukemia can induce the cytoplasmic translocation of the receptor [22].

CRABP2 transports RA from the cytoplasm to the nucleus, promoting RAR ligation and RXR heterodimer formation [12]. Cytoplasmic CRABP2 functions, when coupled with RA, include transportation of RA to different cellular components and metabolization or sequestration of RA in the cytoplasm. However, knowledge about CRABP2 activity unassociated with RA is sparse [23]. Among cytoplasmic CRABP2 interactions, a connection was described with the RNA-binding protein HuR, involved in RNA stability control [24]. Upregulation of CRABP2 has been reported in the blastema of nephroblastomas during the investigation of genes related to nephrogenesis. Nuclear negativity of CRABP2 has been described in 5.31% and cytoplasmic negativity in 6.48% of blastema samples [2]. However, in the samples studied here, including three histological components of nephroblastomas (blastema, epithelium, and stroma), we observed cytoplasmic and nuclear positivity in 100% and 56% of the samples, respectively.

Gupta et al. described the overexpression of CRABP2 in nephroblastoma samples with favorable histology, with increased expression in advanced cancer stages (stage III/IV). The findings of these authors indicate the role of CRABP2 in cell migration and invasion in nephroblastomas [16]. When comparing clinical-pathological prognostic factors to values ​​obtained in quantitative and semiquantitative analyses, we observed increased expression of RARA and CRABP2 in patients with metastasis.

Among our samples, protein expression of RARA was increased in samples from patients undergoing pre-surgical chemotherapy. Increased expression of genes in the RA pathway has been described by other authors in nephroblastoma patients treated with pre-surgical chemotherapy relative to that in patients undergoing surgery as the initial treatment [7]. This phenomenon may be associated with tissue damage and the inflammatory process caused by chemotherapy, which induce increased expression of the RA signaling pathway [25]. Both RARA and CRABP2 immunoexpression appeared to be unrelated with clinicopathological variables, such as local stage, risk group and lymph node status.

In summary, the immunoexpression of RARA and CRABP2 was increased in samples from patients subjected to pre-surgical chemotherapy and in samples from patients with metastatic disease, respectively.

## Conclusions

In conclusion, semiquantitative and quantitative analyses of the markers RARA and CRABP2 indicate these proteins as potential biomarkers of tumor progression and their participation in nephroblastoma tumorigenesis. Complementary studies are needed to better understand the mechanisms involved.

## Additional file

Additional file 1: Figure S1. Immunohistochemical evaluation of RARA and CRABP2 in nephroblastoma and renal parenchyma. RARA immunoexpression: A, renal parenchyma; B, nephroblastoma. CRABP2 immunoexpression: C, renal parenchyma; D, nephroblastoma (40×). (JPEG 3337 kb)

## Abbreviations

CRABP2: Cellular retinoic acid-binding protein 2
DAB: Diaminobenzidine
HMF: Half-magnification field
RA: Retinoic acid
RARA: Retinoic acid receptor alpha
TIFF: Tagged image file format
TMA: Tissue microarrays
