# Supplementary figures and images for: Tissue expression of retinoic acid receptor alpha and CRABP2 in metastatic nephroblastomas
Source: Diagn Pathol. 2018 Jan 22;13:9. doi: 10.1186/s13000-018-0686-z (PMC6389245; doi:10.1186/s13000-018-0686-z)

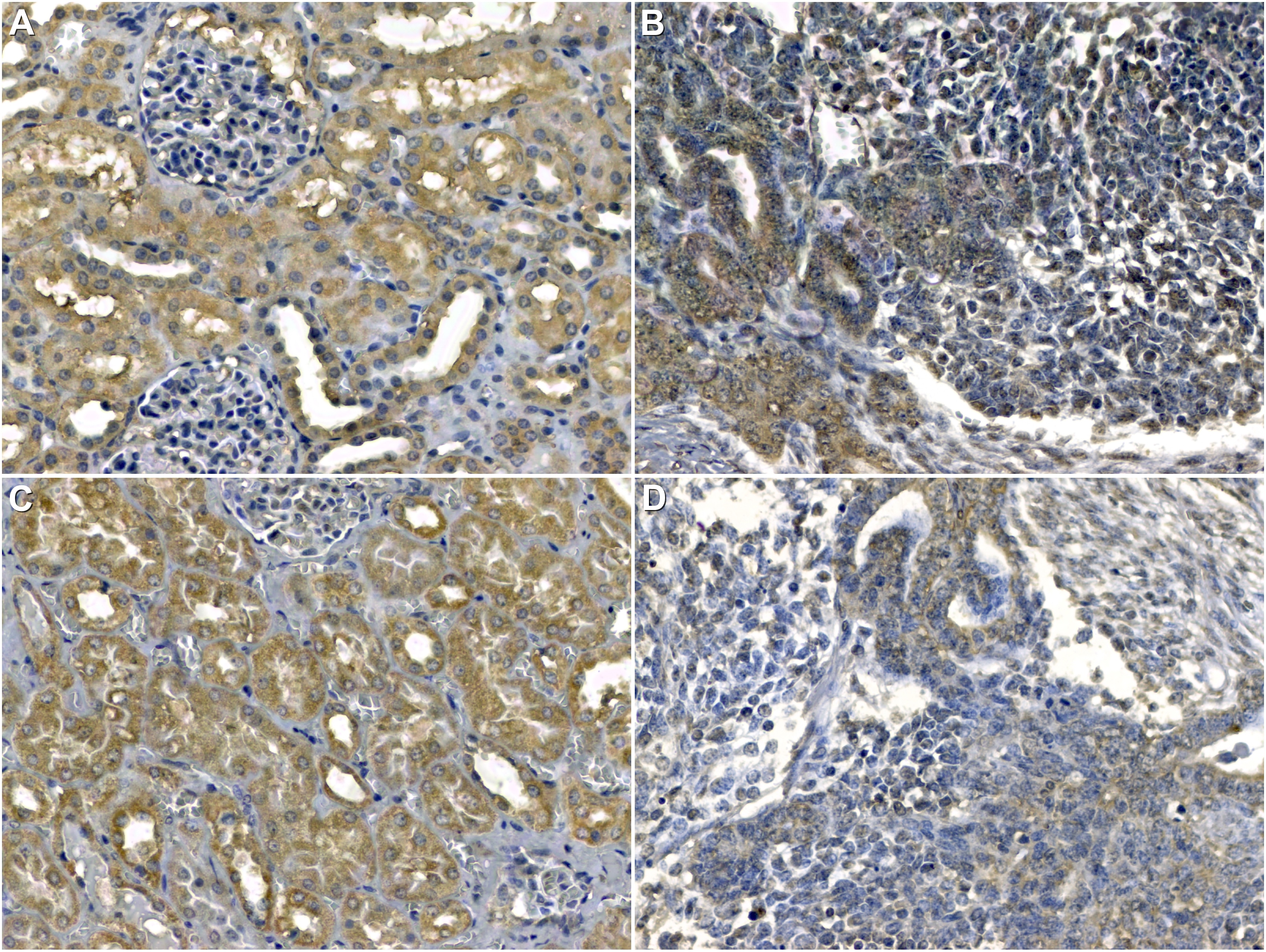

Supplement: Supplementary file 1 — Immunohistochemical evaluation of RARA and CRABP2 in nephroblastoma and renal parenchyma. RARA immunoexpression: A, renal parenchyma; B, nephroblastoma. CRABP2 immunoexpression: C, renal parenchyma; D, nephroblastoma (40×). (JPEG 3337 kb) [file 13000_2018_686_MOESM1_ESM.jpg]
